# Single-cell RNA sequencing reveals the local cell landscape in mouse epididymal initial segment during aging

**DOI:** 10.1186/s12979-023-00345-9

**Published:** 2023-05-11

**Authors:** Jintao Zhuang, Xiangping Li, Jiahui Yao, Xiangzhou Sun, Jiumin Liu, Hua Nie, Yang Hu, Xiangan Tu, Huang Liu, Weibing Qin, Yun Xie

**Affiliations:** 1 Department of Urology and Andrology, The First Affiliated Hospital, Sun Yat-sen University, Guangzhou, 510080 China; 2 Department of Urology, Guangdong Provincial People’s Hospital, Guangdong Academy of Medical Sciences, Southern Medical University, Guangzhou, 510080 China; 3 NHC Key Laboratory of Male Reproduction and Genetics, Guangdong Provincial Reproductive Science Institute, Guangdong Provincial Fertility Hospital, Human Sperm Bank of Guangdong Province, Guangzhou, 510600 China

**Keywords:** Epididymis, Initial segment, Reproductive aging, Single-cell RNA sequencing

## Abstract

**Background:**

Morphological and functional alterations in aging reproductive organs result in decreased male fertility. The epididymis functions as the transition region for post-testicular sperm maturation. And we have previously demonstrated that the epididymal initial segment (IS), a region of the reproductive tract essential for sperm maturation and capacitation, undergoes considerable histological changes and chronic immune activation in mice during aging. However, the local aging-associated cellular and molecular changes in the aged epididymal IS are poorly understood.

**Results:**

We conducted single-cell RNA sequencing analysis on the epididymal IS of young (3-month-old) and old (21-month-old) mice. In total, 10,027 cells from the epididymal IS tissues of young and old mice were obtained and annotated. The cell composition, including the expansion of a principal cell subtype and *Ms4a4b*^Hi^*Ms4a6b*^Hi^ T cells, changed with age. Aged principal cells displayed multiple functional gene expression changes associated with acrosome reaction and sperm maturation, suggesting an asynchronous process of sperm activation and maturation during epididymal transit. Meanwhile, aging-related altered pathways in immune cells, especially the “cell chemotaxis” in *Cx3cr1*^Hi^ epididymal dendritic cells (eDCs), were identified. The monocyte-specific expression of chemokine *Ccl8* increased with age in eDCs. And the aged epididymal IS showed increased inflammatory cell infiltration and cytokine secretion. Furthermore, cell–cell communication analysis indicated that age increased inflammatory signaling in the epididymal IS.

**Conclusion:**

Contrary to the general pattern of lower immune responses in the male proximal genital tract, we revealed an inflammaging status in mouse epididymal initial segment. These findings will allow future studies to enable the delay of male reproductive aging via immune regulation.

**Supplementary Information:**

The online version contains supplementary material available at 10.1186/s12979-023-00345-9.

## Background

The mammalian epididymis is the most important reproductive organ with a highly convoluted tube in males. During the journey passing through the epididymis, immotile testis-derived sperm undergo profound changes to be primed for subsequent fertilization, also known as epididymal transit [1]. This process includes the acquisition of functional molecules, the disposal of excess parts of sperm, and interactions with immune cells for tolerance. The morphologies and distributions of cell types and the epididymis function differ along its septa-separated segments, including the initial segment, caput, corpus and cauda epididymidis. To characterize cellular and molecular features, single-cell RNA sequencing (scRNA-seq) has been performed on different epididymis segments [2, 3]. Notably, the proximal region of epididymis——the initial segment (IS) plays a critical role in this post-testicular sperm processing “pipeline” system [4, 5]. The IS receives the richest blood supply across the epididymis [6, 7]. Gene disruption-induced loss of mouse IS development causes abnormal sperm maturation and male infertility [5]. Compared with other segments, the IS has more diverse cellular components including several epithelial cells (principal, basal, and narrow cells) and considerable immune cell populations [2, 3, 5]. A putative type of principal cell that releases epididymosomes is believed to be rich in IS. Meanwhile, narrow or clear cells responsible for H^+^ ion transport exist exclusively within the IS [5]. Many IS-specific sperm-binding proteins (e.g., Ovch2 and Lcn6) are essential for sperm maturation and subsequent fertilization [8]. Moreover, proximal epididymal sperm are capable of supporting full embryonic development and offspring birth [9], suggesting that the epididymal IS is important for male fertility.

Aging is a degenerative process induced by cellular lesion accumulation, leading to organ dysfunction. Male reproductive aging is one of the main features of the aging process [10]. Advanced age among males negatively affects sperm concentration, motility, morphological rate, reproductive outcomes, and offspring health [11, 12]. However, the complex epididymal anatomy makes the analysis of epididymal aging difficult. And the underlying molecular mechanisms of age-related changes in the epididymis are largely unknown. In order to provide insight into the process of aging in the epididymis, we previously employed bulk RNA sequencing to analyze changes in gene expression with age in the mouse initial segment, caput, corpus, and cauda epididymidis. We reported considerable age-related histological and transcriptomic alterations in the IS compared with those in other segments [7]. And the aging alterations of proximal epididymis were found to be associated with increased immune response–associated pathways [7].

Here, we aimed to characterize the aging-associated cellular and molecular features of the IS of mouse epididymis. The heterogeneous cell populations in the IS and their cell type-specific transcriptome changes are compared between young (3-month-old) and old (21-month-old) mice. We revealed dysregulation of multiple biological processes in aged mouse proximal epdidymis. Notably, the initial segment of the epididymis showed characteristics of inflammaging, with increased infiltration of immune cells and increased proinflammatory molecule expression profiles.

## Results

### scRNA-seq identified diverse cell types in the epididymal IS from young and old mice

**Fig. 1:**
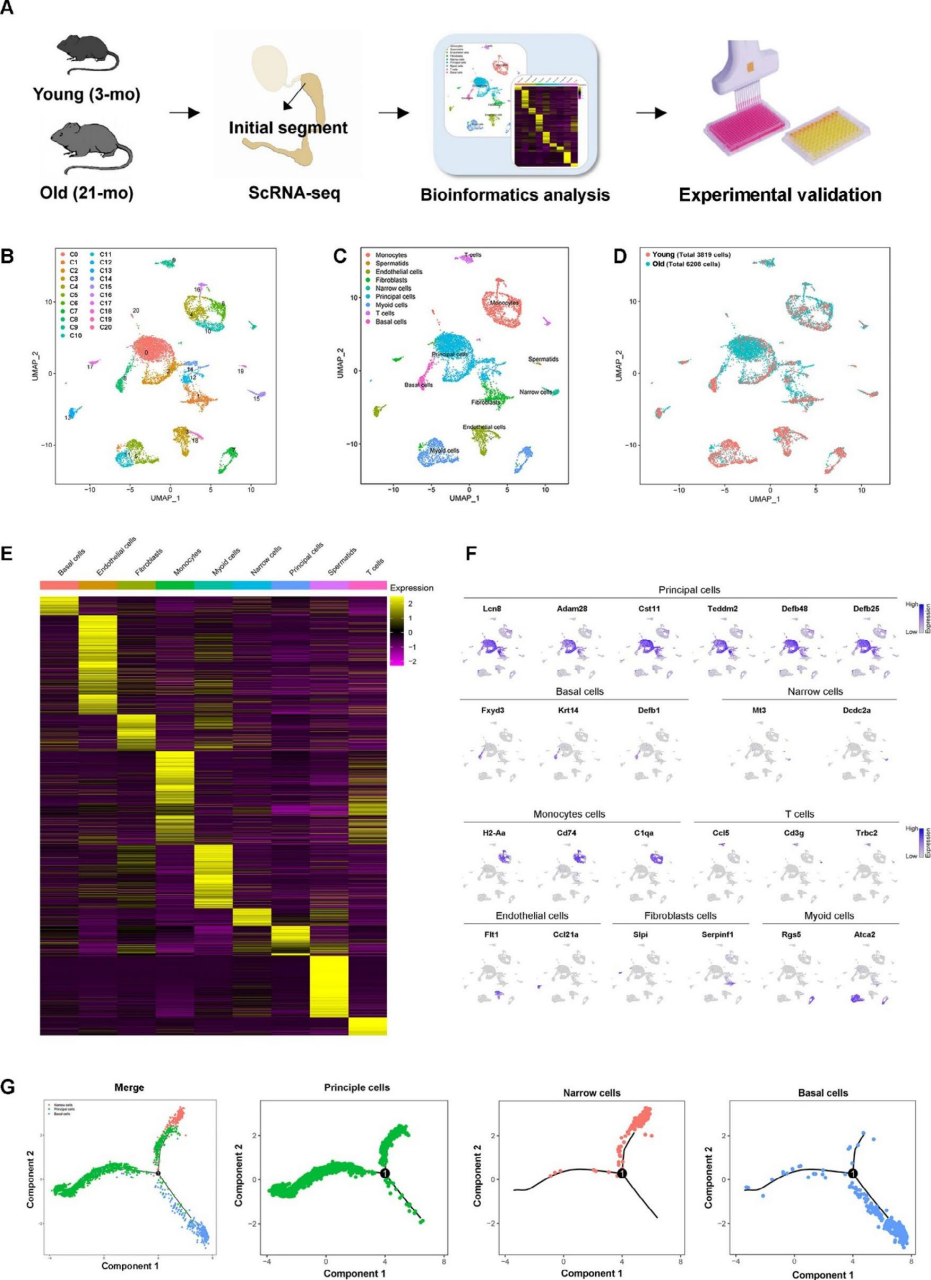
Single-cell transcriptomes of the epididymal initial segment of 3 and 21-month-old mice. (**A**) Flow chart of single-cell RNA sequencing. (**B**) Uniform Manifold Approximation and Projection for Dimension Reduction (UMAP) plot of single cells colored by cluster identity, annotated cell types (**C**), and cell distribution according to ages (**D**). **E**. Differentially expressed genes (DEGs) of cell types. **F**. Expression of key marker genes. **G**. Pseudotime analysis of epididymal epithelial cell transcriptomes

**Fig. 2:**
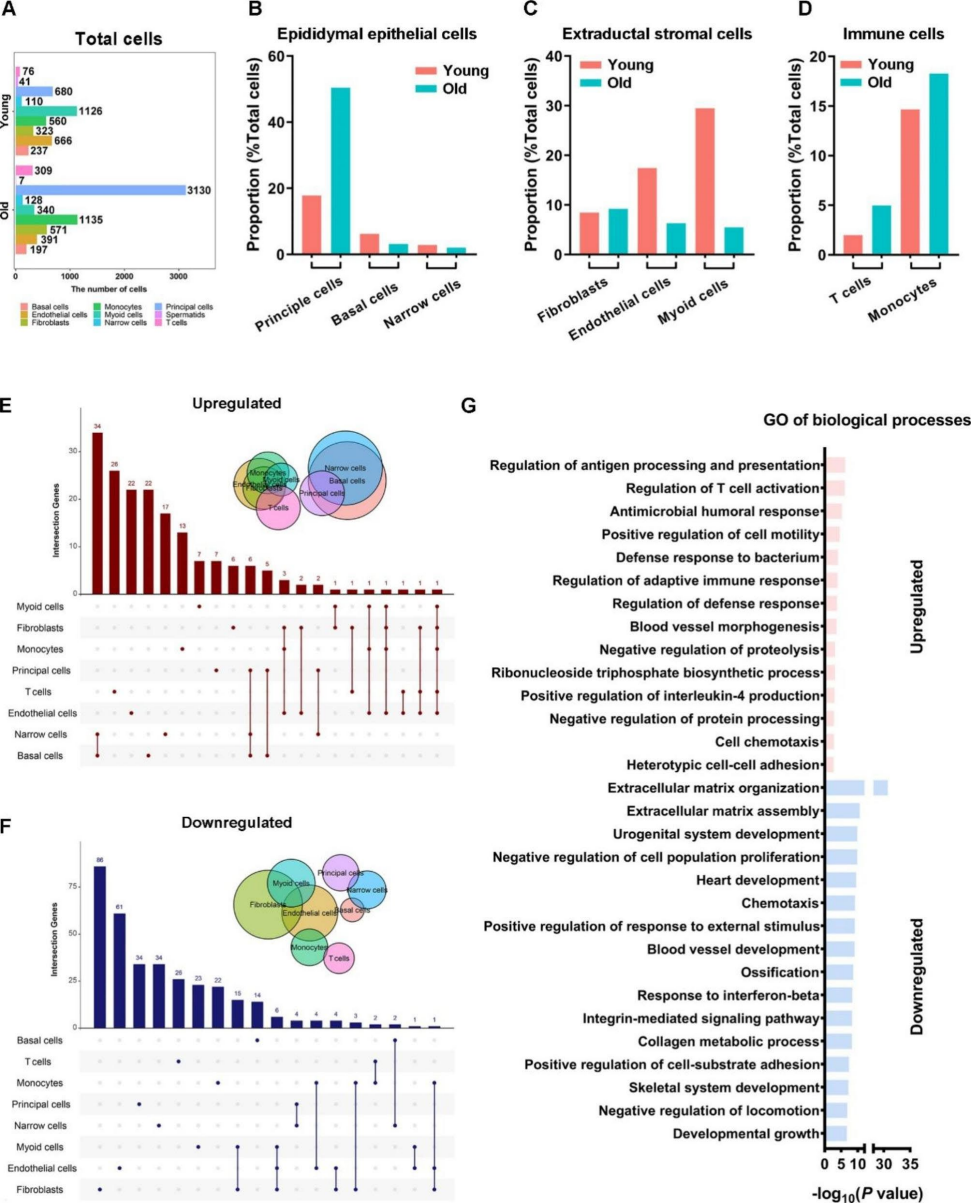
Age alters epididymal cell type proportions and gene expression. (**A**) The number of cells captured by single-cell sequencing in the epididymal initial segment of mice aged 3 and 21 months. Relative proportions of specified cell types of epithelial cells (**B**), extraductal stroma cells (**C**), and immune cells (**D**) among total cells. E. Distribution of differentially expressed genes (DEGs) with upregulated (**E**) and downregulated (**F**) expression in different cell types in young versus aged mice. **G**. Gene Ontology terms of DEGs with overall upregulated and downregulated expression

We performed scRNA-seq on young (3-month-old) and aged (21-month-old) mice using the 10× Chromium platform (Fig. 1A). Because of the small size of IS (< 10 mg/mouse), we obtained mixed tissues rather than independently tagged samples. After quality control and removal of doublets, 10,027 individual cells (3819 and 6208 cells from young and aged mice, respectively) were filtered with 2604 unique molecular identifier (UMI) counts, 1160 expressed genes, and 4.83% mitochondrial genes per cell (Figs. S1A-B). They were divided into 20 clusters using Seurat unsupervised clustering analysis (Fig. 1B). Nine cell types were identified based on typical and recently reported marker genes, including principal cells, basal cells, narrow cells, monocytes, T cells, endothelial cells, fibroblasts, myoid cells, and spermatids/spermatozoa (Fig. 1 C and D). B lymphocytes were not detected, consistent with histological and scRNA-seq analyses of rodents [2, 13]. Furthermore, 645 differentially expressed genes (DEGs) with a fold change of log2 transformed UMI > 1 for each cell type were identified (Fig. 2F and Supplementary Table 1). Fig. S1C shows the GO analysis of DEGs in these cell types. The expression pattern of generally accepted marker genes was projected on Uniform Manifold Approximation and Projection for Dimension Reduction (UMAP) plots, consistent with the annotated cell distribution (Fig. 1F and C).

The cell groups of epididymal epithelial cells (Figs. S2A and B) were analyzed. Principal, narrow, and basal cells were distinguished by classical markers and newly identified gene expression markers (Fig. S2C). GO analysis of the DEGs showed their functions in each epithelial cell, consistent with current knowledge (Fig. S2D). Clusters 0, 2, 12, and 14 (C0, C2, C12, and C14) were identified as principal cells with high expression of lipocalin 8 (*Lcn8*), a disintegrin and metalloprotease 28 (*Adam28*), cystatin 11 (*Cst11*), and *Cst12*, which are previously reported IS-specific genes [7]. The principal cell C0 was the most abundant in the epididymis (Fig. 1A). C0, C2, and C12 showed expression signatures similar to C14 (Fig. S2E), whereas C14, which accounted for a small proportion, showed relatively higher expression levels of *Lcn12*, *Lcn5*, and clusterin (Fig. S2C), which were previously identified as caput-specific genes that initiated expression since the distal IS (or segment 2 in the 10-segment division of mouse epididymis) [7, 14, 15]. These principal cells may represent the transitional proportion of the distal IS and caput epididymis. A previous scRNA-seq analysis of mouse epididymis found that principal cell subgroups possessed distinct segmental specificity, especially from the anatomical perspective of the caput, corpus, and cauda epididymis. Thus, the abovementioned epithelial cells suggested a more complex epithelial system within the IS.

To explore the renewal and differentiation models of the IS epithelium, we performed a pseudotime analysis of all epithelial cells using Monocle 2.0 [16]. This revealed a bifurcating developmental trajectory with a basal cell population splitting into two terminal branches of principal and narrow cells (Fig. 1G). A set of key fate-determining genes was also identified based on the significant gene expression states and pseudotime (Fig. S3). Several of these genes were related to epididymal epithelial development, such as *Adam28*, *Adam7*, *Ovch2*, *Lcn8*, and *Lcn9* [8, 17, 18], and showed increased expression with pseudotime. This corresponds to a previous model of cell regeneration and differentiation in rat and human epididymis that basal cells contain a stem cell pool for principal and narrow cell renewal [4, 19].

### Aging results in increased immune and principal cells and transcriptome alterations

We assessed the differences between eight cell types and gene expression between the ISs of young and aged mice. We observed prevalent alterations in the numbers of epididymal epithelial, stromal, and immune cells (cells obtained per 4 mice) during aging (Fig. 2A). The number of principal and T cells increased with fold changes > 4, and that of monocytes almost doubled, and endothelial and myoid cells decreased in aged mice (Fig. 2A). Basal cells remained consistent in number (Fig. 2A). The number of obtained spermatozoa decreased; however, they did not belong to the structural epididymal cells. Confounding factors during sample processing might affect the number of identified cells; thus, we calculated the proportions/frequencies of cell types in the total epididymal cells [20, 21]. The frequencies of principal cells, T cells, and monocytes increased, whereas those of basal, endothelial, and myoid cells decreased with age (Fig. 2B-D).

Overall gene expression differences were detected in eight epididymal cell types, except for spermatids/spermatozoa. Genes with > 1.5 fold-change (upregulated or downregulated) and *P* < 0.05 were defined as age-related DEGs (A-DEGs) (Fig. 2E and F). The narrow and basal cells showed 34 common genes with upregulated expression within the epididymal epithelium, whereas the fibroblasts, myoid cells, and endothelial cells showed some common genes with downregulated expression within the stromal compartment (Fig. 2E and F), suggesting that the changes because of aging among these cells may be relatively similar. However, few DEGs appeared commonly in transcriptome changes of other cell types (Fig. 2E and F), indicating a cell type-specific aging pattern of IS. GO analysis of the overall age-related DEGs showed that the upregulated transcripts were primarily associated with biological process (BP) terms like “regulation of T cell proliferation”, “antigen processing and presentation of exogenous peptide antigen” and “inflammatory response” whereas the downregulated genes were associated with “positive regulation of response to external stimulus” and “extracellular matrix (ECM) assembly” (Fig. 2G). Our scRNA-seq provides an overview of transcriptome changes during IS aging, consistent with our previous bulk RNA-seq analysis [7]. However, age-related transcriptome differences were mild, with a limited number of A-DEGs (Fig. 2E and F), which may be attributed to the heterogeneity within each cell group and has been analyzed in detail below.

### Aged epididymal IS displays functional and structural degeneration

**Fig. 3:**
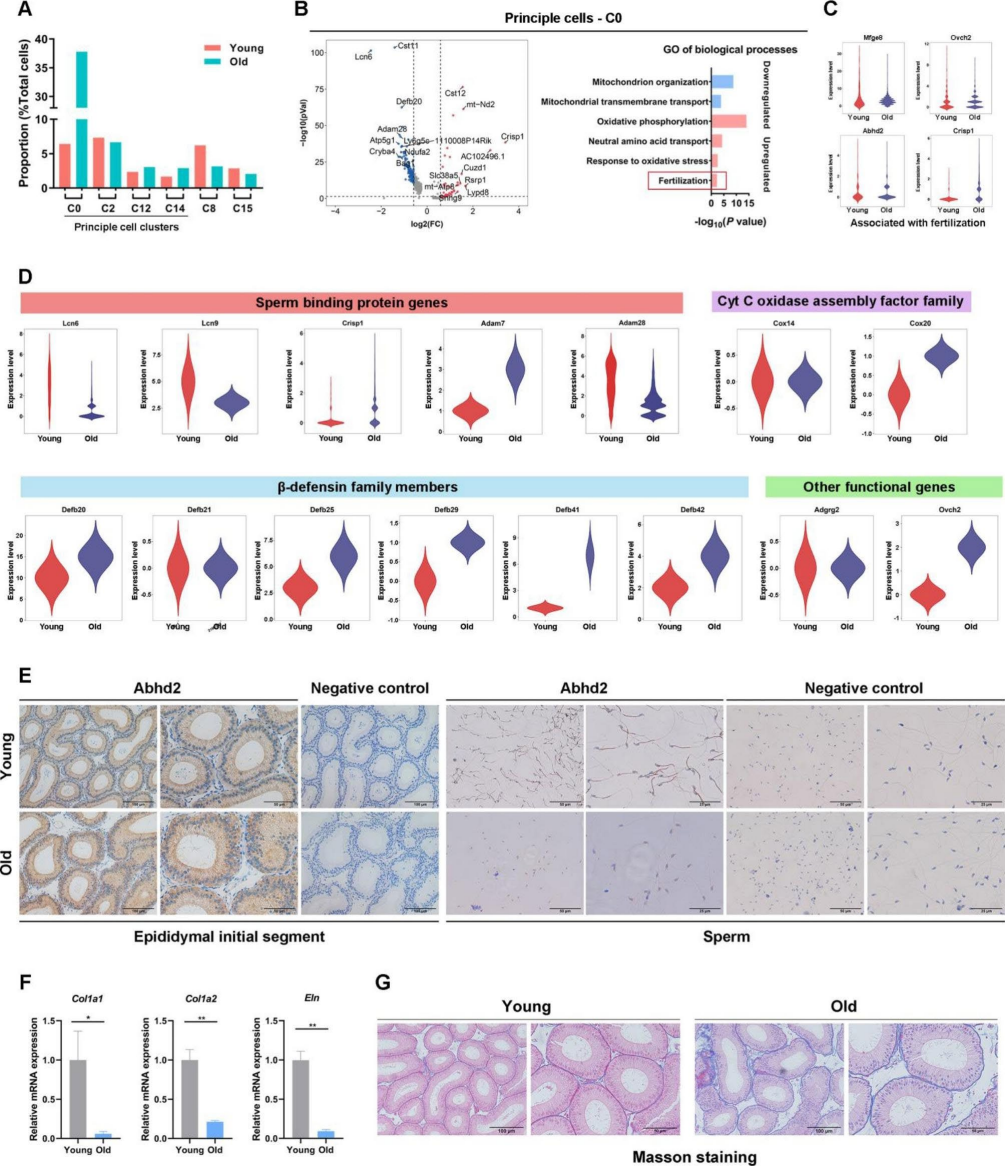
Aged epididymal IS displays functional and structural degeneration. (**A**) Relative proportions of epithelial cell subclusters. (**B**) Funnel plot shows the genes with downregulated and upregulated expression in the principal cells during aging (Left panel). The top 10 genes with upregulated and downregulated expression are marked. Gene Ontology terms of differentially expressed genes with upregulated expression in principal cell C0 are shown in the right panel. (**C**) Representative genes related to the biological process of “fertilization”. (**D**) Violin plots show the expression of epididymal function-related genes. (**E**) Immunohistochemical staining of Abhd2 in the epididymal initial segment and sperm of 3 and 21-month-old mice. (**F**) Expression of *Col1a*, *Col2a*, and *Eln* verified by quantitative real-time polymerase chain reaction verification (n = 6). (**G**) Masson staining of total collagen in the epididymal initial segment of 3 and 21-month-old mice

Principal cells maintain the physiological function of the epididymal epithelium and are the most abundant in the epididymis; therefore, we focused on these cells. We analyzed the cell proportions of four subgroups of principal cells in the two mice age groups. C0 accounted for the highest proportion of epithelial cells, and the expansion of this subgroup resulted in an increased overall number of principal cells (Fig. 3A), suggesting that these cells are more likely to be senescent with age. The cellular functions of C0 were primarily related to BPs of “defensive response to bacteria” and “killing of cells of other organisms” (Fig. S2D), indicating that these functions may be enhanced or activated in aged patients with IS.

We identified 333 and 666 A-DEGs with upregulated and downregulated expression, respectively, in C0 principal cells (Fig. 3B). Surprisingly, GO enrichment analysis revealed that the upregulated gene sets were associated with the GO term “fertilization” (Fig. 3B, the left panel), represented by *Abhd2*, *Crisp1*, *Ovch2*, and *Mfeg8*. *Abhd2* is a well-known gene related to sperm capacitation that activates the sperm calcium channel CatSper and acrosome reaction (AR) [22]. *Abhd2* showed IS-enriched expression based on our previous RNA-seq data [7]. *Crisp1* and *Mfeg8* are two sperm-binding protein genes important for sperm maturation, AR, and consequential sperm-egg fusion [23, 24]. *Ovch2* is required for the processing and sperm-fertilizing ability of *Adam3* [8].

Other functional genes also underwent age-related changes in C0 expression (Fig. 3D). For example, *Lcn6*, *Lcn9*, and *Adam7*, three sperm-binding protein genes that are required for the integrity of the epididymal epithelium and normal sperm maturation capacity [18, 25], showed decreased expression with age (Fig. 3D). The expression of Cytochrome C oxidase assembly factor subunit members (e.g., *Cox*14, 20, 6b1, 7c) were generally reduced in C0 with age. These were important mitochondrial structural genes (33,008,142) and enriched BPs of “oxidative phosphorylation.” The transcripts of a series of β-defensin family member genes (e.g., Defb20, 21, 25, 29, 41, 42) were also downregulated with age in C0 (Fig. 3D). These are primary molecules secreted by principal cells to resist bacterial infection [26]. *Adgrg2*, an X-linked congenital bilateral absence of the Vas Deferens gene involved in regulating the epididymal fluid reabsorption process[27], showed increased expression in C0 with age (Fig. 3D). Overall, the principal cells displayed a state of sperm overactivation and multiple key functional gene expression changes.

Three types of extraductal stromal cells, including fibroblasts, myoid cells, and vascular/lymphatic endothelial cells, were annotated (Figs. S4A-B). Two clusters (C1 and C17) of fibroblasts showed highly expressed DEGs enriched to BP terms such as “extracellular matrix organization”; myoid cells with three clusters (C5, C7, and C11) showed highly expressed DEGs related to “muscle system process” and another three clusters (3, 13, and 18) constituted endothelial cells that were functionally related to “regulation of vascular development” (Figs. S4C and D). These functions are consistent with our current understanding of these cells. Although the transcriptome changes induced by aging were modest in these stromal cells (Figs. S5A-C), they exhibited decreased expression levels of a series of ECM-related genes in the IS, including collagen family genes (*e.g., Col1a1* and *Col1a2*), elastin (*Eln*), fibrillin family genes (*e.g., Fbn1* and *Fbn5*), and several genes essential for formation and maintenance of ECMs (e.g., *Serpinh1* and *Mmp2*) (Fig. S5D). This indicated widespread degenerative changes in the extraductal microenvironment and could reasonably explain the loose arrangement of aged epididymal ducts previously observed [7].

We conducted immunohistochemical staining of the epididymal IS and spermatozoa for *Abhd2*, which showed increased expression and binding levels, respectively (Fig. 3E, left panel). Interestingly, the *Abhd2* protein was predominantly distributed in the sperm tail and sperm head of young and old mice, respectively (Fig. 3E, right panel), suggesting that old mice sperm might be asynchronously activated along with delayed maturation in the proximal epididymis. Using qPCR, we verified that the expression of *Col1a1*, *Col1a2*, and *Eln* was significantly downregulated with advancing age in the epididymal IS (Fig. 3F). The IS tissues of young and old mice were analyzed with Masson’s staining. Epididymal ducts showed significant fibrosis and collagen deposition in the basement membrane and interstitium (Fig. 3G). Overall, the aged epididymal IS displays functional and structural degeneration.

### Aged epididymal dendritic cells show transcriptome signatures related to increased chemotactic pathways

Five cell clusters (C16, 4, 6, 10, and 20) were identified as monocytes (Figs. S5A and B). Except for C10, the proportions of C16, C4, C6, and C20 of the captured cells generally increased (Fig. S6C). These five monocyte subgroups showed relatively similar functional features based on GO BP terms, including “antigen presentation” and “leukocyte migration” (Fig. S6D). C10 and C4 accounted for a considerable proportion of the monocyte cluster and highly expressed chemokine receptor gene *Cx3cr1*, a marker for previously characterized epididymal dendritic cells (eDCs). eDCs were exclusively found in the IS and responsible for sperm immune tolerance and the removal of apoptotic epithelial cells [28–30]. Few monocytes in C20 may represent classical dendritic cells with highly expressed DC marker genes *S100a9*, *S100a8*, *Il1b*, and *Plac8* (Fig. S6E). C4, C6, and C10 were also highly expressed complement component genes (e.g., *C1qa* and *C1qb*) (Fig. S5E), indicating their role in autoimmunity and inflammation [31, 32]. Some monocyte clusters may also contain adult macrophages, indicated by MHC II subunit gene expression (e.g., *CD74*, *H2-Aa*, and *H2-Eb1*) (Fig. S6E). However, these clusters usually lacked distinctive transcriptome features, revealing heterogeneity.

**Fig. 4:**
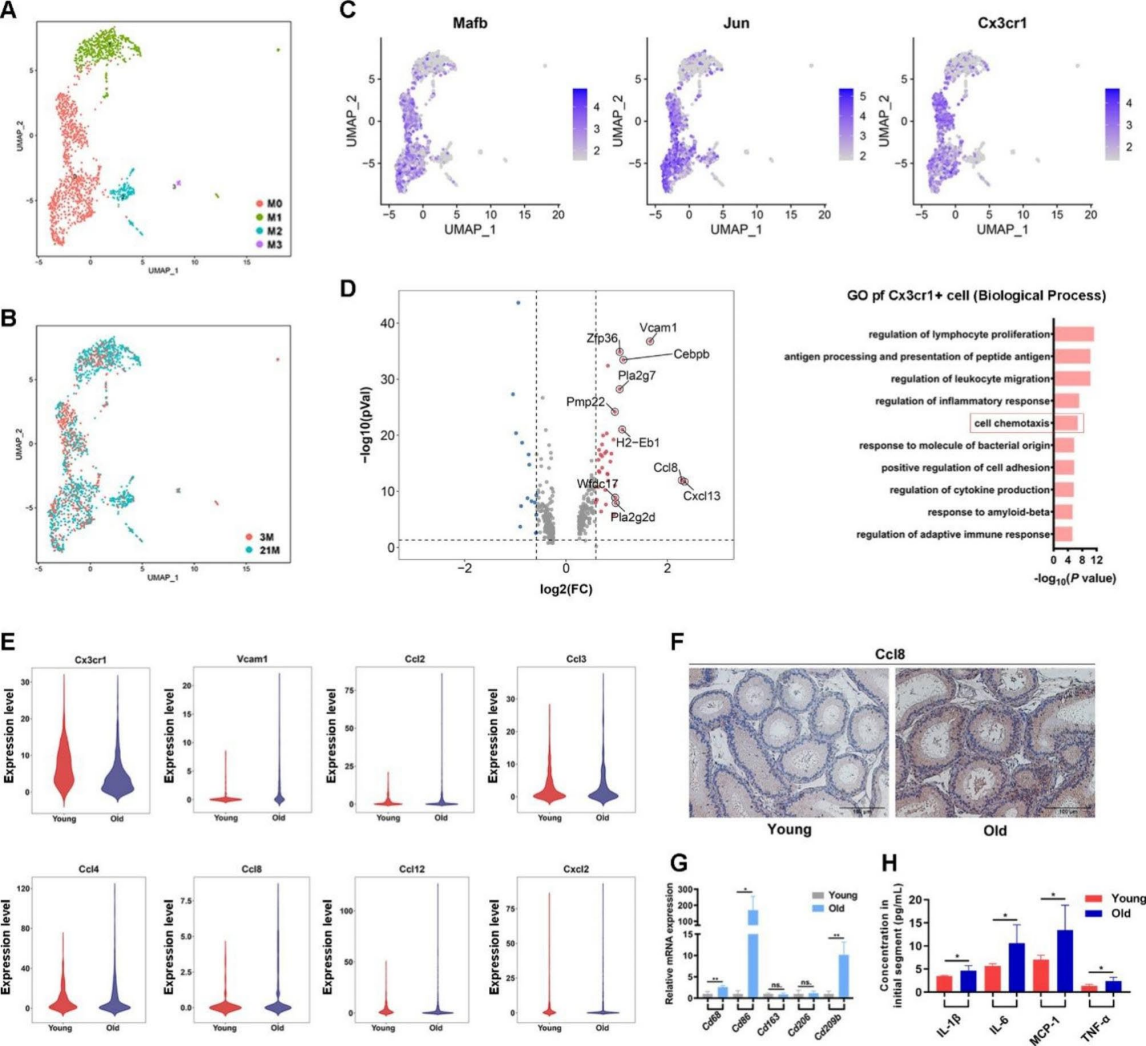
Aging-dependent alterations in monocytes. **A**. Uniform Manifold Approximation and Projection for Dimension Reduction (UMAP) plot of monocyte subclusters and the cell distribution in 3- and 21-month-old mouse samples (**B**). **C**. Expression of key marker genes of monocyte subcluster 0 (M0). **D**. Funnel plot shows the genes with downregulated and upregulated expression in the principal cells during aging (left panel). The top 10 genes with upregulated expression are marked. Gene Ontology terms of differentially expressed genes with upregulated expression in M0 are shown in the right panel. **E**. Representative genes related to the biological process of “Cell chemotaxis”. **F**. Immunohistochemical staining of Ccl8 in the epididymal initial segment of young and old mice. **G**. Expression of monocyte markers evaluated by quantitative real-time polymerase chain reaction verification (n = 6). **H**. Inflammatory cytokines with significantly increased levels determined by antibody chip (n = 4)

To analyze the intragroup heterogeneity of monocytes, we reclustered all 1695 monocytes into four subgroups: M0, M1, M2, and M3 (Fig. 4A and B). M0 was characterized by the high expression of marker genes *Cx3cr1*, *Mafb*, and *Jun* (Fig. 4C). This group was considered the eDCs having less heterogeneity. M0 also accounted for the majority of the monocytes in the IS, consistent with a previous report on eDCs [28]. M3 represented a typical dendritic cell group, characterized by high expression of marker genes *Xcr1*, *Clec9a*, and *Itgae* (*CD103*), whereas the remaining clusters M1 and 2 were not classified (Fig. 4A and B).

We subsequently analyzed the transcriptome differences of eDCs in the young and old epididymal IS (Fig. 4D). The eDCs were proportionally consistent with age (Fig. S6F). The A-DEGs of M0 with upregulated expression were functionally related to BPs of “cell chemotaxis” and “lymphocyte proliferation” represented by chemokine *Ccl8*, adhesion factor *Vcam1*, and T cell activation regulator *Pla2g2d* (Fig. 4D). According to our previous bulk RNA-seq data, these genes also revealed a common upregulation pattern during aging at the bulk tissue level [7]. *Ccl8* showed the largest fold change. Its protein product displays chemotactic activity toward monocytes and lymphocytes at inflammatory sites. We performed qPCR to verify the expression patterns of these genes with age (Fig. 4E). The increased expression of *Ccl8* during aging was validated by immunohistochemistry (Fig. 4F). To evaluate the immune status of the epididymal IS, we compared the expression levels of classic monocyte markers in young and old IS tissues using qPCR and detected the levels of 18 pro-inflammatory factors using an inflammatory factor microarray chip. The expression levels of pro-inflammatory macrophage marker genes *CD86* and *CD209b* were found to significantly increase in old mice (Fig. 4G), suggesting that IS monocytes were polarized toward a pro-inflammatory state. The microarray chip detected that the levels of four pro-inflammatory cytokines, IL-1β, IL-6, TNF-α, and MCP-1, increased significantly with age (Fig. 4H).

### T cells showed aging-related activation states

**Fig. 5:**
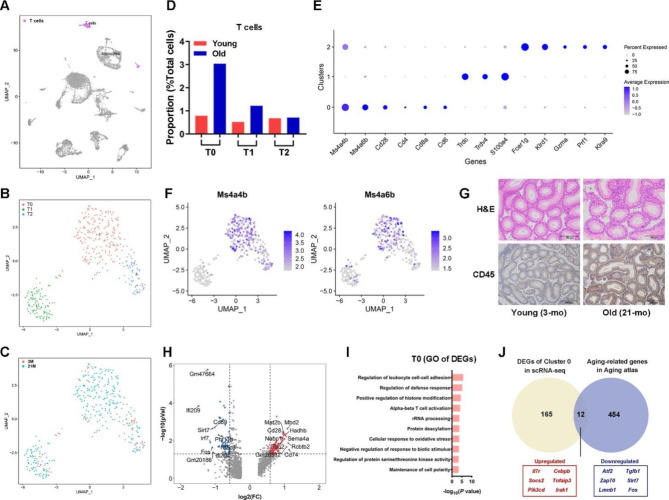
Aging-dependent alterations in T cells. **A**. Uniform Manifold Approximation and Projection for Dimension Reduction (UMAP)plot of T cells, subclusters (**B**), and the cell distribution in 3 months and 21 months old samples (**C**). **D**. Alterations in cell proportions of T cell subclusters. **E**. Expression patterns of marker genes used for cell type annotation. The fraction of cells that expressed the marker genes is indicated by the size of the circle, and the means of the expression levels of marker genes are indicated by the color. **F**. Cell markers of T0. **G**. Immunohistochemical staining for CD45 in the epididymal initial segment of young and old mice. **H**. Funnel plot shows the genes with downregulated and upregulated expression in the principal cells during aging. The top 10 genes with upregulated and downregulated expression are marked. **I**. Gene Ontology terms of differentially expressed genes with upregulated expression in T0. **J**. Overlapping genes with downregulated or upregulated expression in the aged T0 subtype and included in the Aging atlas database

In total, 385 cells from cluster 9 were identified as T cells with the typical expression of *Cd3d*, *Ccl5*, and *Trbc2* (Figs. 1B and 5A). We reclustered the T cell population to yield three subclusters (T0, T1, and T2) (Fig. 5B and C). The substantial increase in T0 cell numbers accounted for the major age-related increase in T cells (Fig. 5D). None of the T cell clusters showed a clear association with classical CD4^+^ and CD8^+^ T cell polarization, except for a few *CD4*^Hi^ and *CD8*^Hi^ T cells in T0 (Fig. 5E). Most T0 cluster cells were characterized by the expression of MS4A family member genes, including *Ms4a4b* and *Ms4a6b* (Fig. 5E and F), reported to be involved in T cell activation regulation and autoantigen response [33]. Subcluster T1 may represent a γ&δ T cell population with high expression levels of *Trdc*, *Trdv4*, and *Tmemb176a/b*, which was also believed to react with autologous cells [34–36]. Moreover, the subcluster T2 coexpressed natural killer cell marker genes (e.g., *Fcer1g*, *Klrd1*, and *Klra9*) and T cell marker genes (e.g., *Cd3d* and *Ccl5*) (Fig. 5E), potentially representing a group of natural killer T (NKT) cells [37]. Our findings altogether showed that the epididymal IS contains various T cell subtypes. The expression of the lymphocyte pan-maker CD45 in the epididymal IS increased with age, validating the aggregation of T cells with age (Fig. 5G).

Considering the dramatic increase in T0 during aging IS, we further analyzed the transcriptome differences of T0 subcluster cells with age (Fig. 5H). GO enrichment analysis showed that the age-related DEGs of T0 with upregulated expression were related to BP terms of immune activation pathways, including the “regulation of leukocyte cell-cell adhesion”, “regulation of defense response” and “cellular response to oxidative stress” (Fig. 5I). The expression of genes related to GO terms of “positive regulation of histone modification” was upregulated (Fig. 5I), consistent with the understanding of age-related T cell epigenetic changes [38]. We compared the A-DEGs of T0 cells with the aging atlas database [39]. We screened 12 aging-related genes, including six and six genes with upregulated and downregulated expression, respectively (Fig. 5J). These genes may be key factors or biomarkers for epididymal T cell senescence.

### Inflammatory signaling increases in the epididymal IS of the elderly

**Fig. 6:**
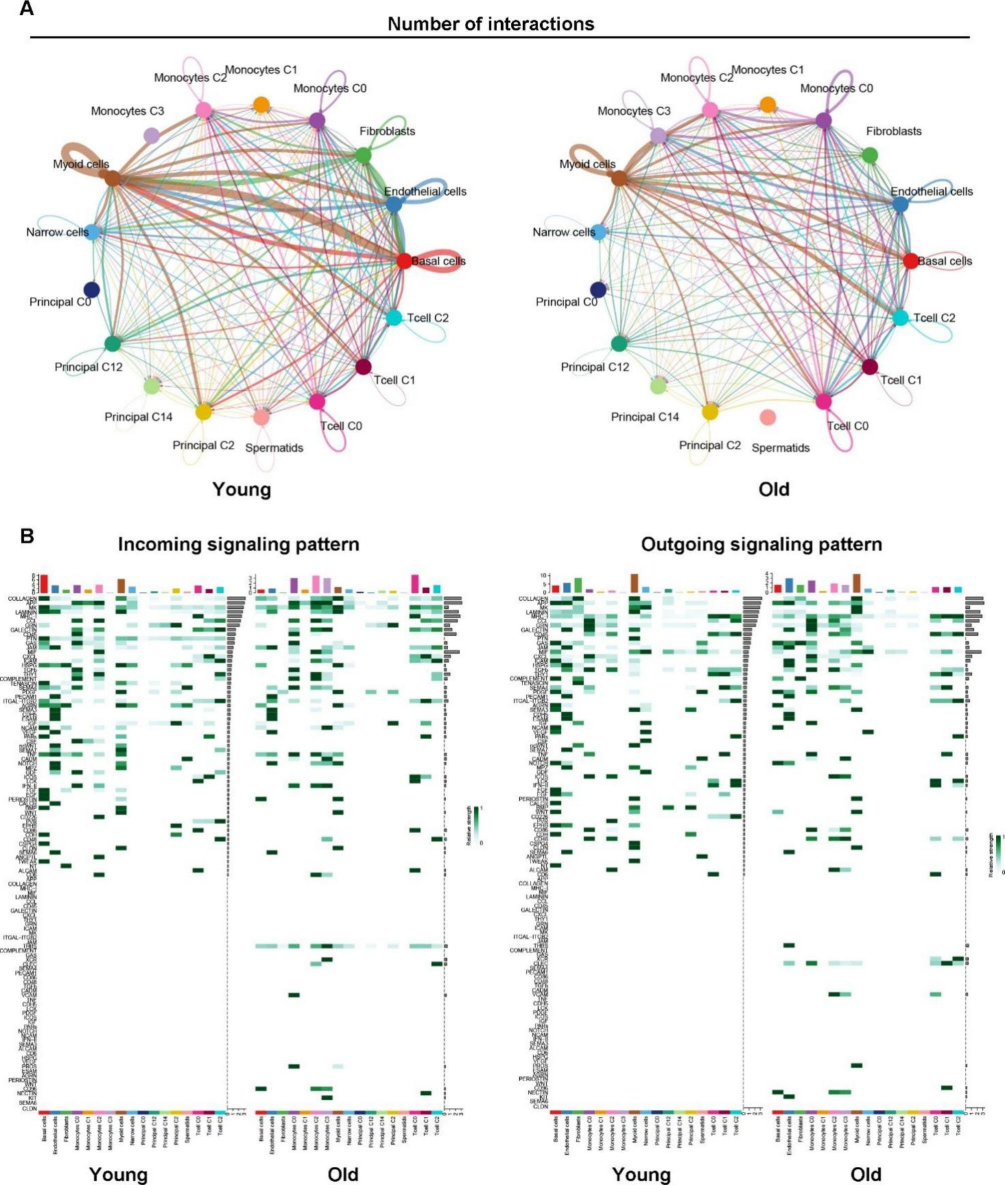
Predicted cell–cell communication network in the epididymal initial segment of young and old mice. (**A**) The number of interactions is shown by the boldness of the line. (**B**) Predicted regulatory signaling. The number of receptors and ligands is shown in colored cells and subclusters are shown by the column diagram

The epididymal IS plays a crucial role in sperm maturation by coordinating interactions between spermatozoa, epithelial cells, stromal cells, and immune cells [40]. To evaluate the complex interactions between cells in the epididymal IS of young and old mice, we delineated signaling pathways composed of ligand–receptor interactions between various types of cells using the cell–cell communication database. In the epididymal IS of young mice, communication between myoid and basal cells accounted for a certain proportion (Fig. 6A and B). Considering the close spatial arrangement of these two cell types, myoid cells have potentially regulated basal cells as a stem cell pool for epithelial renewal. Fibroblasts were also important to signal initiators in young mice, acting with myoid, endothelial, and basal cells (Fig. 6A). In old mice, the interactions between monocyte subclusters, especially C0 and T cell subclusters, increased (Fig. 6A, right panel). The weight of eDCs (or monocytes M0) and T0 subcluster cells, as signal initiators and receivers, respectively, increased (Fig. 6A). The weight of monocyte self-regulation signaling molecules also increased, which may be attributed to the “autocrine” immune cell mechanism to attract more immune cells using dominant cell chemotactic signaling molecules, such as CCLs (Fig. 6B). MIF-related incoming and outgoing signaling was significantly upregulated in multiple cell types in old mice (Fig. 6B). These results imply increased inflammatory pathways in the aged epididymal IS.

## Discussion

Studies on humans and rodents have shown that age has a progressive effect on male fertility [41]. Much attention has been paid to the effects of aging on the testes [21, 42]. From a clinical perspective, asthenospermia is the most common manifestation of abnormal semen parameters compared with sperm concentration/count, normal morphology rate, or semen volume in elderly men [43, 44]. Decreased sperm motility, instead of other abnormal semen parameters, significantly affects assisted reproductive technology outcomes in elderly infertile men [45, 46]. Sperm-fertilizing capacity can be compromised with age as evidenced by the negative effects of paternal age on time to conceive or assisted reproductive technology outcomes using donor eggs [47, 48]. These observational studies provide substantial evidence of the susceptibility of the epididymis to aging, which impairs the process of sperm maturation. However, no molecular explanation has been established for age-related changes in the epididymis.

**Fig. 7:**
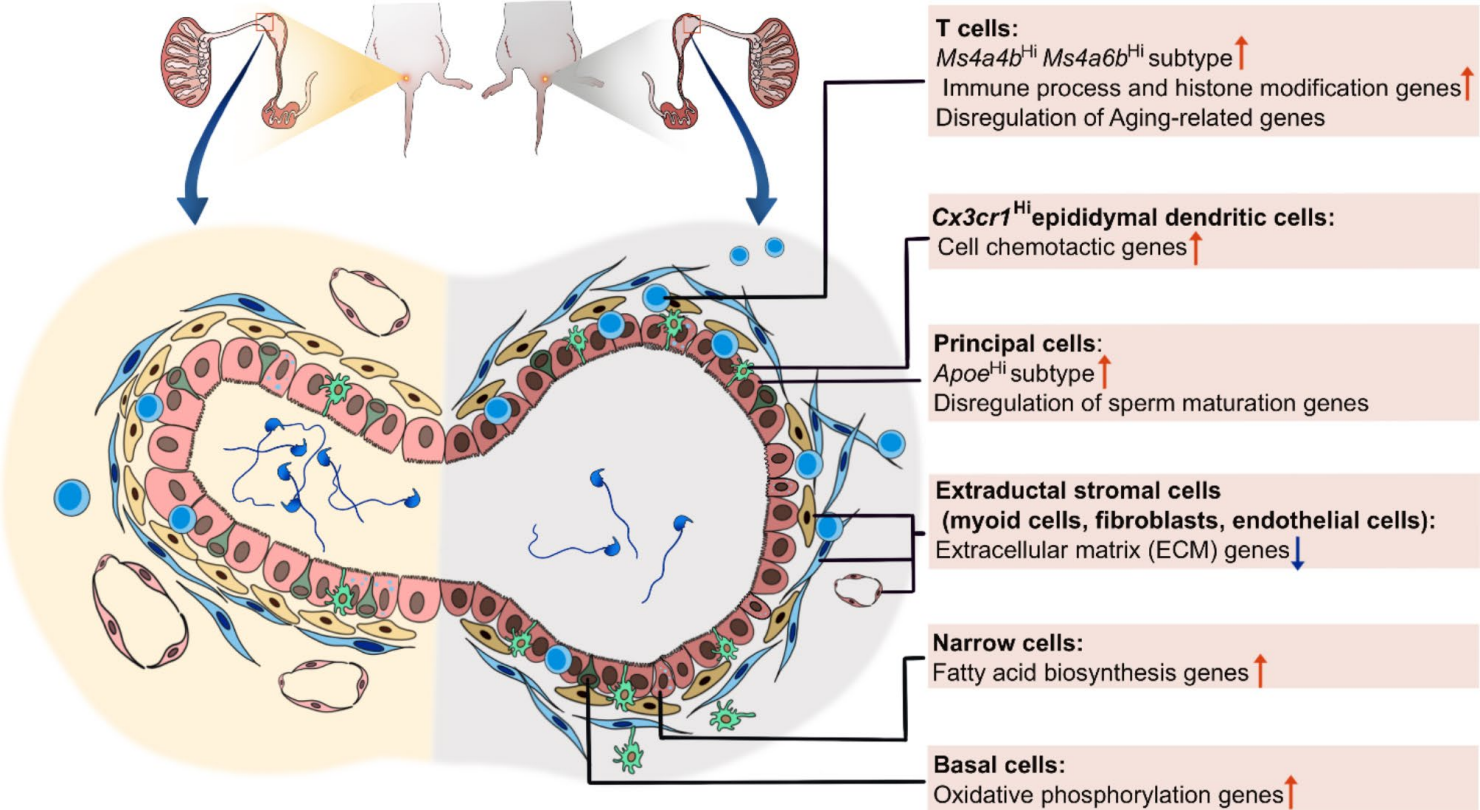
Single-cell RNA sequencing of epididymal initial segment in young and old mice. Age-related changes at cell and transcriptome levels provide evidence suggesting sperm maturation associated functional deterioration, stromal degeneration, and an inflammaging status occurred in the proximal epididymis

Based on our previous histological and RNA-seq analysis of aging changes in the male reproductive regions of mice, the epididymis showed region-dependent age-related changes, especially in the epididymal IS [7]. Therefore, herein, we provide a single-cell view of the molecular changes in the epididymal IS during aging (Fig. 7). Epididymal IS participates in sperm maturation [40]. The proximal epididymis may act as a downstream sperm quality controller through the disposal of defective sperm [49]. Herein, the aging epididymal IS showed an increase in the number and frequency of principal and immune cells as captured by the scRNA sequencing technique, which was consistent with a previous histological study [13]. Altered expression of multiple epididymal functional genes was observed in principal cells with age, including several sperm-binding proteins (e.g., *Lcn6, Adam7*, and *Crisp1*) and the epididymal fluid reabsorption gene *Adgrg2*. Failed *Adgrg2* expression can increase fluid viscosity and hinder sperm transit [50], suggesting that the sperm transit activity of aged mice is impaired.

Unexpectedly, the expression of genes related to sperm fertilization in the epididymis increased with age, including *Abhd2, Crisp1*, *Ovch2*, and *Mfeg8*. *Abhd2* is a well-known positive regulator of sperm AR. The immunohistochemistry staining of sperm revealed the distribution of Abhd2 protein translocated from the sperm tail in young mice to the head in old mice. Abhd2 is distributed in the human sperm tail [22]. The age-related changes in the distribution of Abhd2 are unclear, which may correspond to different sperm states during AR. These changes indicate asynchrony in AR onset and hyperactivation during sperm capacitation as showed by another study using hamster model [51]. This situation may lead to inappropriate sperm activation in the proximal epididymis, thereby leading to the premature depletion of fertilization capacity in old mice. However, the underlying mechanism remains unclear.

The epididymal immune status is complex and unique. Compared with the blood–testis barrier, the blood–epididymis barrier in the epididymal IS is considered less tight, with considerable local immune cell colonization, including T cells and monocytes [13]. However, the exact cell composition lacks clarification at the histological and cellular levels. The proximal epididymis is in a more immunosuppressive state. The more it extends to the distal epididymis, the more it tends to be immune-activated [30]. Our scRNA-seq analysis detected increased immune cell components in aged IS, including previously characterized and IS-specific *Cx3cr1*^Hi^ eDCs and a T cell subtype that may be responsible for the autoimmune response. Consistent with previous studies, the *Cx3cr1*^Hi^ eDCs are the most abundant immune cell type in the epididymis [28]. The eDCs exclusively present in the IS are responsible for sperm immune tolerance and the removal of apoptotic epithelial cells [28, 30]. Cx3cr1 also participates in the chemotaxis of *Cx3cr1*^+^ mononuclear phagocytes and T cells [52, 53]. Some aging-related genes, including chemokine *Ccl8*, adhesion factor *Vcam1*, and T cell activation regulator *Pla2g2d* increased with age in eDCs. These aging-related genes specifically expressed by monocytes were consistent with our previous bulk RNA sequencing data [7], suggesting that monocytes, especially eDCs, may play a crucial role in initiating T cell activation. Functional enrichment analysis identified the upregulated expression of genes related to multiple immune response pathways in the old group. The IS of the aging epididymis shows characteristics of inflammaging. However, the induction of epididymal immune activation remains to be revealed. Bacterial infection, urine reflux, decreased epididymal barrier function, and the abovementioned immune clearance of defective sperm are possible explanations for age-related immune activation [54, 55].

The relationship between T cells and aging has garnered increasing attention. Aging-related T-cell dysfunction can lead to the failure of immune tolerance mechanisms [38]. In the mouse epididymal IS, we identified the unconventional spectra of T cell populations and their age-related changes. The increase in CD3^Hi^Ms4a4b^Hi^Ms4a6b^Hi^ T cells was the largest age-related cell population. Ms4a family members, including Ms4a4b, play a permissive role in lowering the threshold for antigen-induced T-cell activation [33]. Putative γδ T cell and NKT cell populations were also identified in mouse IS, which represent the innate immune cell populations. These two groups of cells are well-known effector cells in epithelial homeostasis, autoimmunity, and fibrotic diseases [56]. Studies suggest that these unconventional T cells play an important role in aging [38, 57]. Although the number of these two groups increased with age, we did not observe a significant change in the cell proportion, which may be attributed to the small number of T cells. The specific roles of these immune cells in epididymal IS of young and old mice require further study. Targeting these cells to regulate epididymal aging immune status is a possible way to delay male reproductive aging in the future.

## Conclusion

Our study depicted an aging atlas of the IS of the epididymis in a naturally aging mouse model by scRNA-seq. The IS of the epididymis showed characteristics of inflammaging, with increased infiltration of immune cells and increased pro-inflammatory molecule expression profiles, paving the way for possible therapies through immunomodulation. Principal cells showed excessive aging-related sperm activation and disrupted epithelial functions related to sperm maturation. Moreover, the disorders of the ECM caused by stromal cell aging may also be involved in the changes in the aging microenvironment and the cell–cell interaction network.

## Methods

### Animals

All animal experiments adhered to the International Council for Laboratory Animal Science guidelines and the procedures were approved by the Ethics Committee of Sun Yat-sen University (SYSU-IACUC-2022-001904). C57BL/6 mice aged 3 (young) and 21 months (old) were obtained and maintained at the Laboratory Animal Center of Sun Yat-sen University under a 12 h/12 h light/dark cycle with free access to food and water. The mice were euthanized via CO_2_ exposure. Samples were collected between 9 a.m. and 11 a.m. to prevent the potential influence of circadian rhythm.

### Preparation of single-cell suspensions

The mouse IS epididymal tissue was dissected in a 10-cm cell culture dish on ice on a sterilized bench according to a previous study. The ISs were cut into pieces after removing excess fat and blood. Then, 90% Dulbecco’s modified eagle medium with 5 mL of 10% fetal bovine serum, 1 mg/mL of collagenase I, and 0.5 mg/mL of collagenase IV (Gibco, CA, USA) was added for initiating digestion. After incubation at 37℃ for 15 min, 6 mL of ice-cold 1 × phosphate-buffered saline (PBS) was used to terminate the reaction, and a 40-µm cell filter (Millipore, Billerica, MA, USA) was used to remove cell debris. The precipitate was centrifuged (300 *g*, 5 min) and resuspended in 1 mL 1 × PBS. Cell concentration and viability were measured using a Countess cell counter (Thermo Fisher Scientific, Waltham, Massachusetts, USA) and adjusted to 1 × 10^6^/mL cells with 1× PBS.

### Single-cell RNA sequencing

scRNA-seq was performed using a 10 × Genomics™ platform (10x Genomics, Pleasanton, California, USA) according to the manufacturer’s protocol (www.10xgenomics.com). Briefly, each batch of 10,000 single cells of epididymal IS pooled from four young and four old mice was loaded on a microfluidic chip to form the gel bead-in-emulsions reaction system. Constructed libraries were sequenced on an Illumina NovaSeq 6000 sequencing platform (Illumina Inc., San Diego, CA, USA) with a paired-end 150 bp read length.

### Single-cell data acquisition and analysis

Raw sequencing data were aligned and quantified using CellRanger count command (10x Genomics) against mouse reference genome GRCm38 provided by Cell Ranger. Doublets were removed using the scrublet software. The count matrices were further analyzed using Seurat package (version 4.0.1) in R software (version 4.0.3) [58]. For each sample, Cells with a mitochondrial gene ratio > 20% and gene number > 2500 or < 200 were excluded. The top 2,000 highly variable genes were identified using FindVariableFeatures function with the vst method. Batch correction were corrected using the IntegrateData function in the Seurat 4.0 [59]. The integrated Seurat object was subsequently scaled and analyzed by principal component analysis (PCA). The top 30 principal components (PCs) were used to perform the Uniform Manifold Approximation and Projection (UMAP) dimensional reduction and construct a K nearest neighbor(KNN) graph. Clusters were then identified using FindClusters function with the resolution parameter as 0.5. Markers of each cluster were identified using FindAllMarkers function with a Wilcoxon Rank Sum test. Cell types were assigned based on well-known markers.

### Pseudotime analysis

For pseudotime analysis, we used monocle 2 package [16]. In brief, the UMI count matrices and meta.data from narrow, principal and basal cells were used to construct a CellDateSet object. Top 1000 siginificantly differently expressed genes were selected as ordering genes. DDRTree was used to reduce dimension, and all cells were ordered using orderCells function.

### Bioinformatics analysis

The R language-embedded bioinformatics analysis tool Metascape (https://metascape.org/gp/index.html#/main/step1) was used for Gene Ontology (GO) enrichment analysis [60], and R language was used for visualization. GO terms with a *P*-value < 0.01 indicate significant enrichment. The OmicStudio online tool (http://www.omicstudio.cn/) was used to analyze the gene set distribution and draw funnel diagrams. Cell–cell communication was analyzed using CellChat (version 1.1.3).

### Hematoxylin and eosin (H&E) staining and masson staining

The epididymal IS tissues were fixed in 4% paraformaldehyde. After dehydration and paraffin-embedding, 5-µm-thick slices were used for H&E staining. Masson staining was performed using Masson’s trichrome staining solution (G1006, Servicebio, Wuhan, China) according to the manufacturer’s instructions. H&E-stained sections were observed under a microscope (BX53; Olympus, Tokyo, Japan).

### Immunohistochemical staining

For immunohistochemical staining, paraffin-embedded mouse epididymal tissues were dewaxed and rehydrated, and endogenous peroxidase activity was eliminated using 3% H_2_O_2_. After repairing the antigens with 1 × Tris ethylenediaminetetraacetic in a microwave, 0.3% Triton X100 was used to increase the cell membrane permeability for 10 min, followed by incubation with 5% bovine serum albumin at room temperature for 30 min. The first antibody was added, including anti-Lcn6 (LS-C806117, LifeSpan BioSciences, 1:100), anti-Abhd2 (14039-1-AP, Proteintech, 1:100), anti-Ccl8 (BS70757, Boster, 1:100), anti-CD45 (20103-1-AP, Proteintech, 1:100), and horseradish peroxidase-labeled goat anti-rabbit IgG (BA1055, Boster, 1:100) and incubated at 4℃ overnight. The immunostained sections were observed under a microscope (BX53, Olympus, Tokyo, Japan).

### Inflammatory cytokine analysis

Inflammatory cytokines in the epididymal IS from young (n = 4) and old mice (n = 4) were determined using a Milliplex Luminex xMAP inflammatory cytokine antibody chip (Merck, Kenilworth, NJ, USA), and the levels of 18 canonical cytokines were detected (Gm-CSF, interferon-γ, interleukin 1α [IL-1α], IL-1β, IL-2, IL-4, IL-5, IL-6, IL-7, IL-10, IL-12 (P70), IL-13, LIX, IL-17 A, KC, MCP-1, MIP-2, and tumor necrosis factor-α [TNF-α]).

### Quantitative polymerase chain reaction (PCR)

Total RNA was extracted using RNAiso Plus (Takara, Japan) following the manufacturer’s instructions. A cDNA library was constructed using the PrimeScript RT Master Mix Kit (RR036A, Takara, Japan). Quantitative PCR was performed using TB Green Premix Ex Taq II (RR820A; Takara, Japan) according to the manufacturer’s instructions. All primers used (Sangon Biotech, Shanghai, China) are listed in Supplementary Table 2.

### Statistical analysis

Statistical analysis was conducted using the SPSS software (version 26.0; IBM Corp., NY, USA). Quantitative data were presented as mean ± standard deviation obtained from at least four independent experiments. Between-group comparisons were performed using the Student’s *t*-test, and *P* < 0.05 was considered statistically significant.

## Electronic supplementary material

Below is the link to the electronic supplementary material.

**Additional file 1**: **Supplementary Fig. 1** Overall features of single-cell sequencing data. A. Quality control data, including a unique molecular identifier (UMI) count expressed genes and mitochondrial genes ratio in young and old (B) samples. C. The Gene Ontology (GO) terms of differentially expressed genes of each cell type with a fold change of log2 transformed UMI > 1

**Additional file 2**: **Supplementary Fig. 2** Single-cell features of epididymal epithelial cells. A. Uniform Manifold Approximation and Projection for Dimension Reduction (UMAP) plot of epithelial cell populations and their distribution in young and old samples (B). C. Differentially expressed genes (DEGs) of each epithelial cell cluster with a fold change of log2 transformed unique molecular identifier > 1. D. The Gene Ontology (GO) terms of DEGs. E. Expression patterns of principal cell marker genes. The fraction of cells that expressed the marker genes is indicated by the size of the circle, and the means of the expression levels of marker genes are indicated by the color

**Additional file 3**: **Supplementary Fig. 3** The expression pattern of differentially expressed genes of epithelial cells along with pseudotime

**Additional file 4**: **Supplementary Fig. 4** Single-cell features of extraductal stromal cells. A. Uniform Manifold Approximation and Projection for Dimension Reduction (UMAP) plot of stromal cell populations and their distribution in young and old samples (B). C. Differentially expressed genes (DEGs) of each stromal cell cluster with a fold change of log2 transformed unique molecular identifier > 1. D. The Gene Ontology (GO) terms of DEGs

**Additional file 5**: **Supplementary Fig. 5** Disorder of extracellular matrix (ECM)-related gene expression in extraductal stromal cells. A. Funnel plot shows genes with downregulated expression during aging in fibroblasts, myoid cells (B), and endothelial cells (C). The lower panel shows the Gene Ontology terms of the genes with downregulated expression. D. Violin plots show the expression of ECM-related genes

**Additional file 6**: **Supplementary Fig. 6** Single-cell features of monocytes. A. Uniform Manifold Approximation and Projection for Dimension Reduction (UMAP) plot of monocyte populations and their distribution in young and old samples (B). C. Cell proportions of monocyte clusters in young and old samples. D. The Gene Ontology (GO) terms of differentially expressed genes clusters with a fold change of log2 transformed unique molecular identifier > 1. E. Expression patterns of monocyte marker genes. The fraction of cells that expressed the marker genes is indicated by the size of the circle, and the means of the expression of marker genes are indicated by the color. F. Cell proportions of monocyte subclusters in young and old samples

**Additional file 7: Supplementary Table 1** List of the cell markers of cell tyes in the epididymal initial segment

**Additional file 8: Supplementary Table 2** Primers used for the quantitative real-time PCR

## Data Availability

The data reported in this paper have been deposited in the GEO repository (Accession no. GSE226310).

## Abbreviations

Abhd2: Abhydrolase domain containing 2
Adam7: ADAM metallopeptidase domain7
Ccl8: C-C motif chemokine ligand 8
Clec9a: C-type lectin domain containing 9a
Cxcrl: C-X3-C Motif Chemokine Receptor 1
Col1a: Collagen 1a
Crisp1: Cysteine rich secretory protein 1
ECM: extracellular matrix
eDCs: epididymal dendritic cells
Eln: Elastin
Fcer1g: Fc Epsilon Receptor Ig
GM-CSF: Granulocyte-macrophage Colony Stimulating Factor
IS: Initial segment
Itgae: Integrin subunit alpha e
Jun: Jun proto-oncogen
KC: keratinocyte-derived chemokine
Klrd1: Killer cell lectin like receptor d1
Lcn6: Lipocalin 6
LIX: Lipopolysaccharide-induced CXC chemokine
Mafb: MAF BZIP transcription factor b
MCP-1: Monocyte Chemotactic Protein 1
MIP-2: Macrophage Inflammatory Protein 2
Ms4a4b: Membrane-Spanning 4-Domains, Subfamily A, Member 4 A
Ms4a6b: Membrane-Spanning 4-Domains, Subfamily A, Member 6B
Ovch2: Ovochymase 2
Plac8: Placenta associated 8
scRNA-seq: Single-cell RNA sequencing
UMAP: Uniform Manifold Approximation and Projection
UMI: Unique Molecular Identifier
Vcam1: Vascular Cell Adhesion Molecule 1
Xcr1: X-C Motif Chemokine Receptor 1.
