# Supplementary figures and images for: Single-cell RNA sequencing reveals the local cell landscape in mouse epididymal initial segment during aging
Source: Immun Ageing. 2023 May 11;20:21. doi: 10.1186/s12979-023-00345-9 (PMC10173474; doi:10.1186/s12979-023-00345-9)

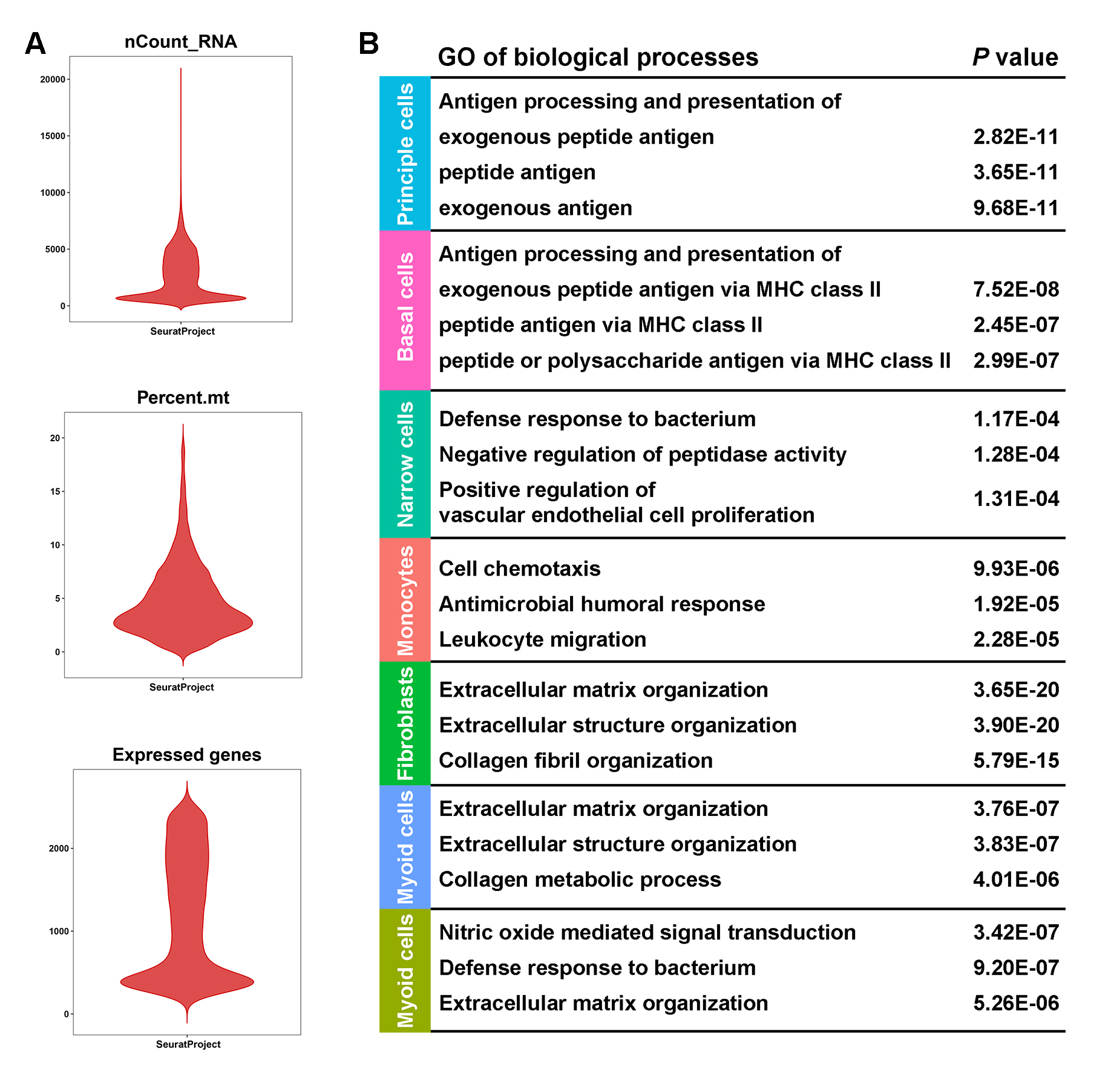

Supplement: Supplementary file 1 — Additional file 1: Supplementary Fig. 1 Overall features of single-cell sequencing data. A. Quality control data, including a unique molecular identifier (UMI) count expressed genes and mitochondrial genes ratio in young and old (B) samples. C. The Gene Ontology (GO) terms of differentially expressed genes of each cell type with a fold change of log2 transformed UMI > 1 [file 12979_2023_345_MOESM1_ESM.jpg]

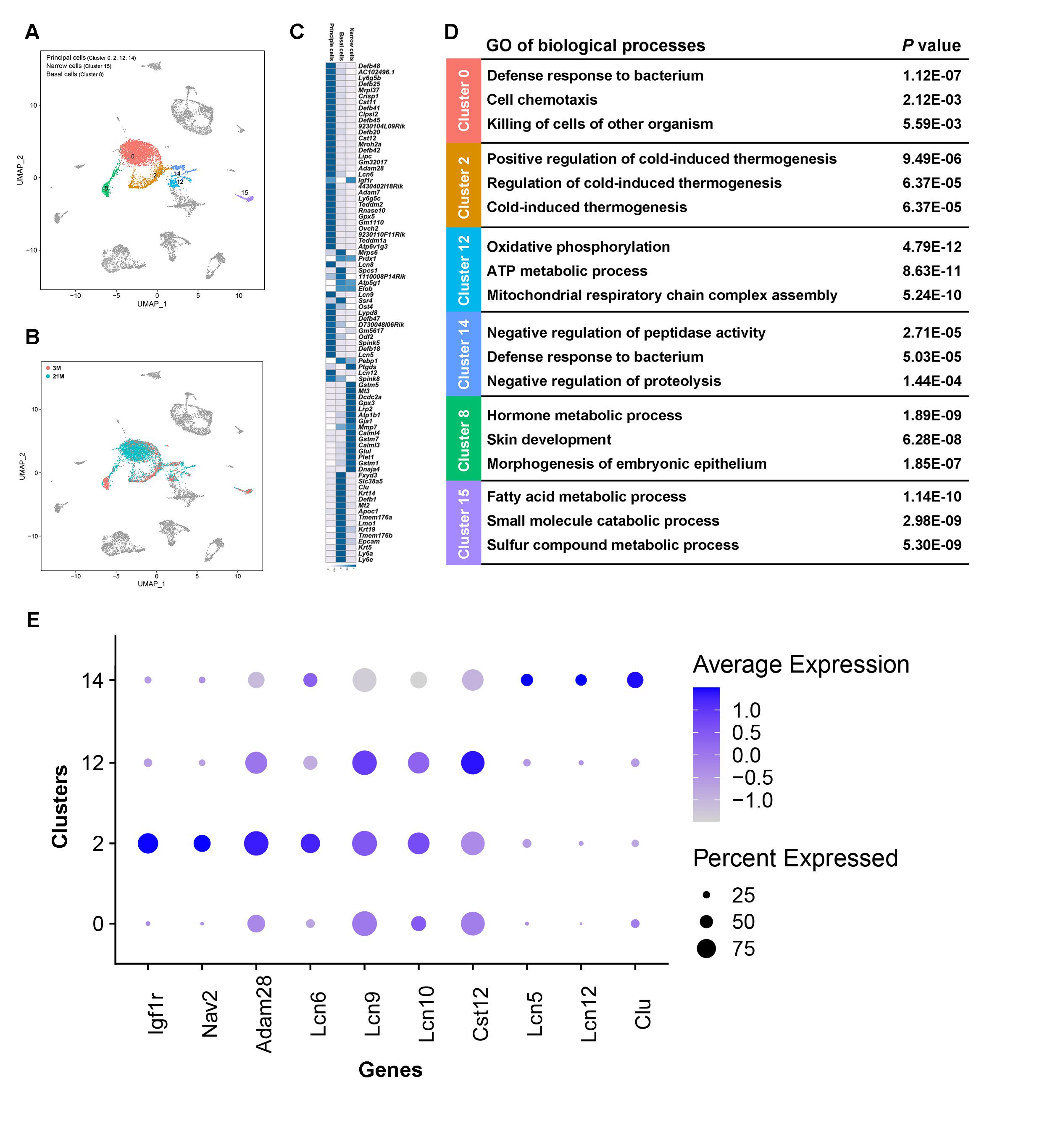

Supplement: Supplementary file 2 — Additional file 2: Supplementary Fig. 2 Single-cell features of epididymal epithelial cells. A. Uniform Manifold Approximation and Projection for Dimension Reduction (UMAP) plot of epithelial cell populations and their distribution in young and old samples (B). C. Differentially expressed genes (DEGs) of each epithelial cell cluster with a fold change of log2 transformed unique molecular identifier > 1. D. The Gene Ontology (GO) terms of DEGs. E. Expression patterns of principal cell marker genes. The fraction of cells that expressed the marker genes is indicated by the size of the circle, and the means of the expression levels of marker genes are indicated by the color [file 12979_2023_345_MOESM2_ESM.jpg]

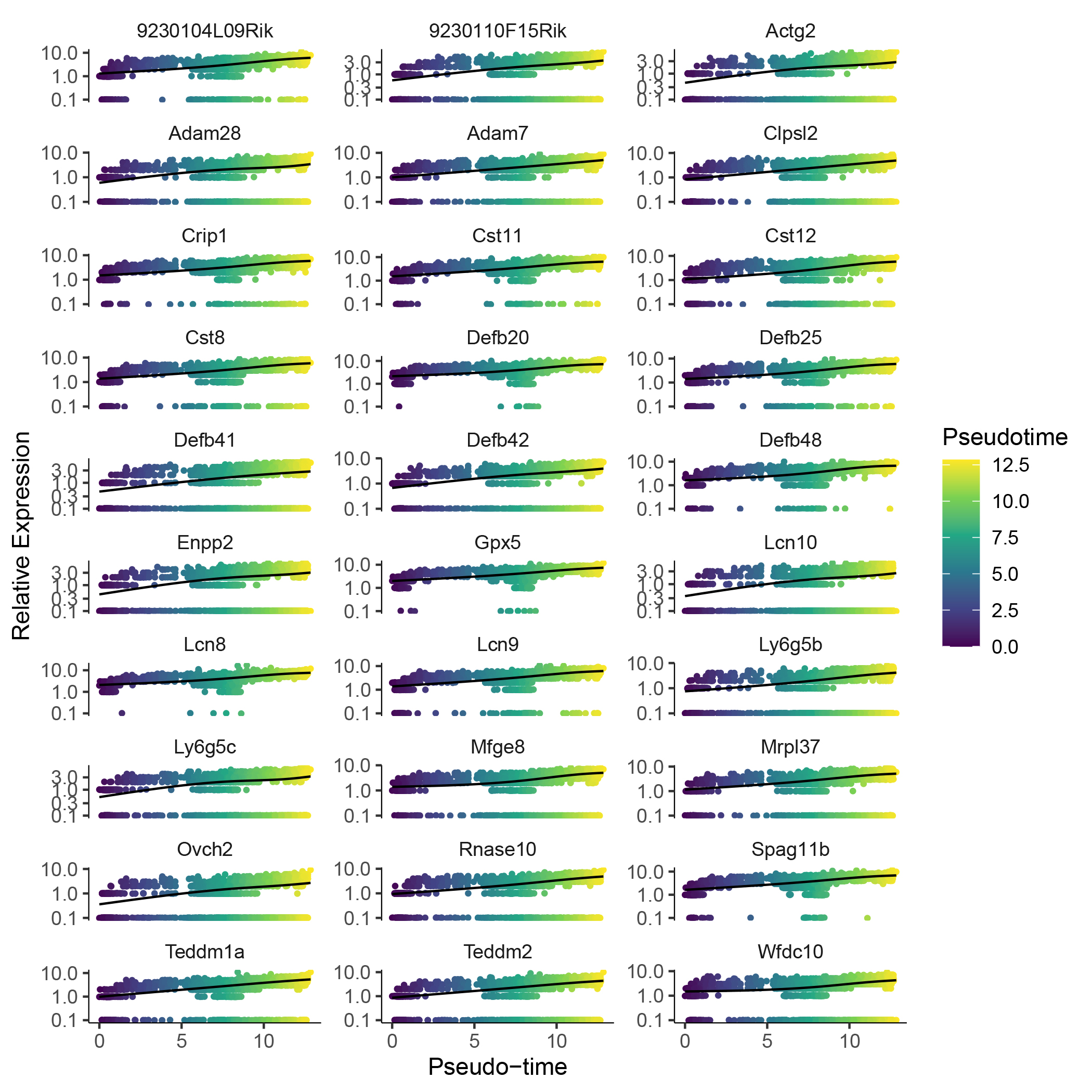

Supplement: Supplementary file 3 — Additional file 3: Supplementary Fig. 3 The expression pattern of differentially expressed genes of epithelial cells along with pseudotime [file 12979_2023_345_MOESM3_ESM.jpg]

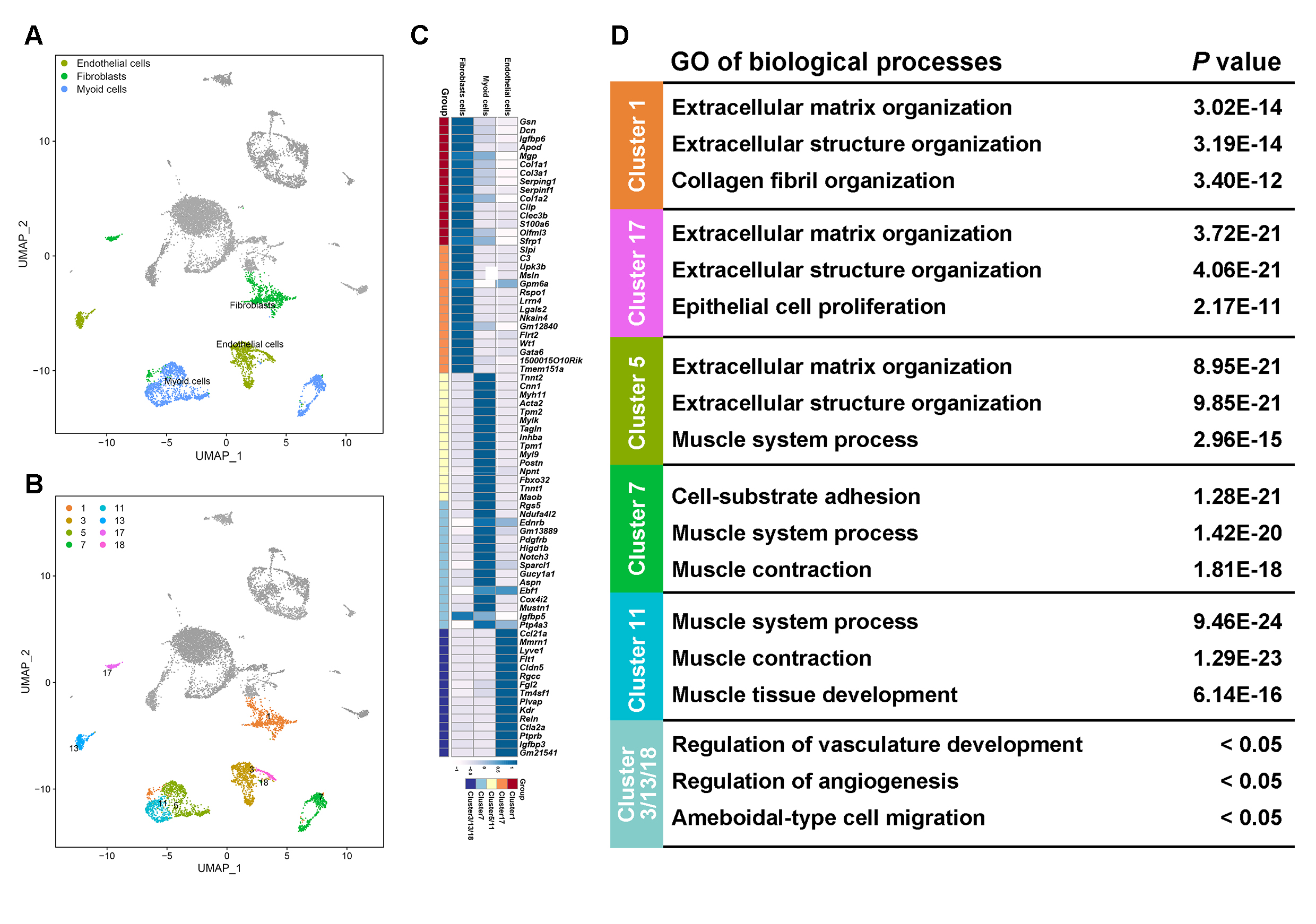

Supplement: Supplementary file 4 — Additional file 4: Supplementary Fig. 4 Single-cell features of extraductal stromal cells. A. Uniform Manifold Approximation and Projection for Dimension Reduction (UMAP) plot of stromal cell populations and their distribution in young and old samples (B). C. Differentially expressed genes (DEGs) of each stromal cell cluster with a fold change of log2 transformed unique molecular identifier > 1. D. The Gene Ontology (GO) terms of DEGs [file 12979_2023_345_MOESM4_ESM.jpg]

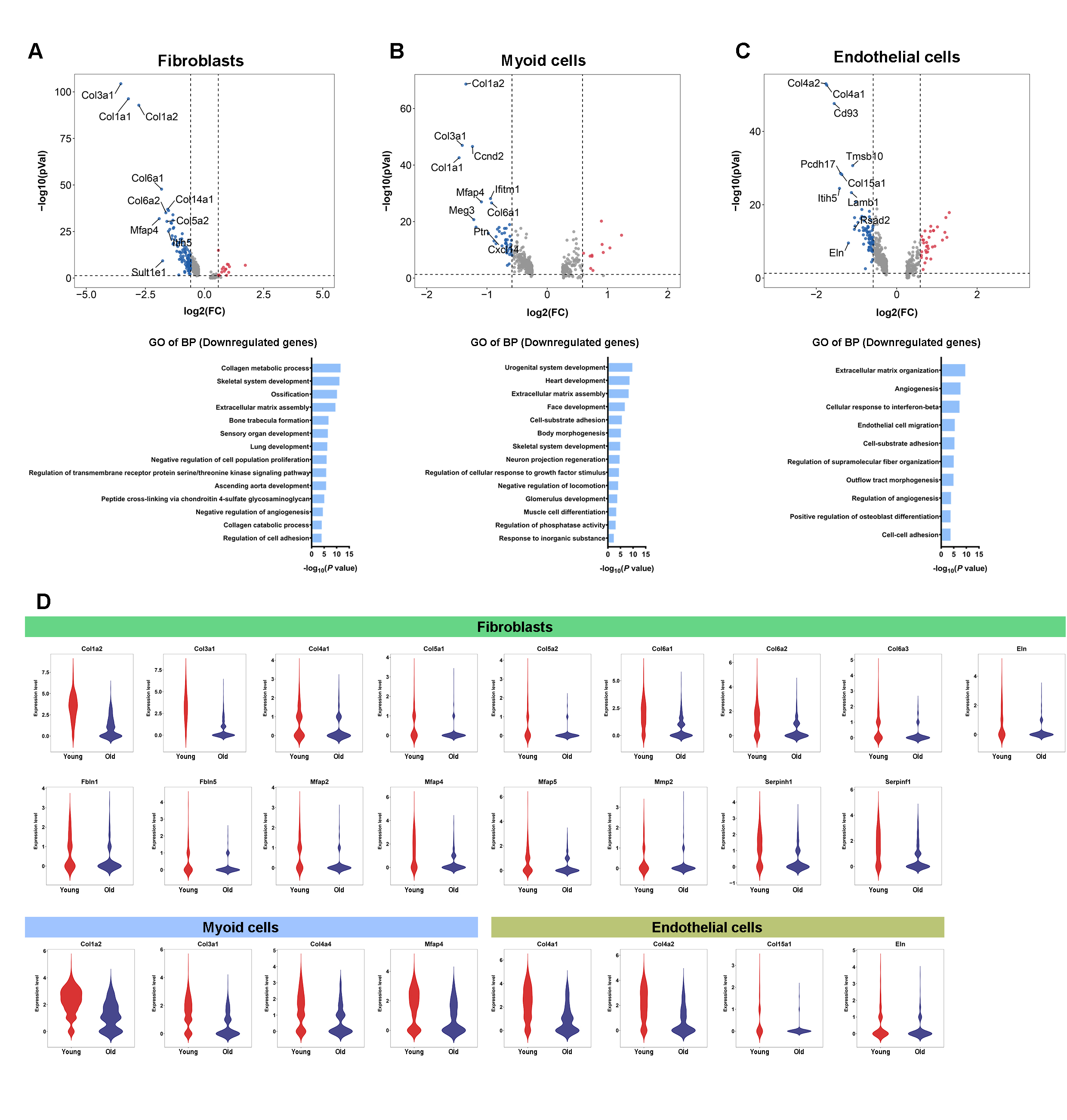

Supplement: Supplementary file 5 — Additional file 5: Supplementary Fig. 5 Disorder of extracellular matrix (ECM)-related gene expression in extraductal stromal cells. A. Funnel plot shows genes with downregulated expression during aging in fibroblasts, myoid cells (B), and endothelial cells (C). The lower panel shows the Gene Ontology terms of the genes with downregulated expression. D. Violin plots show the expression of ECM-related genes [file 12979_2023_345_MOESM5_ESM.jpg]

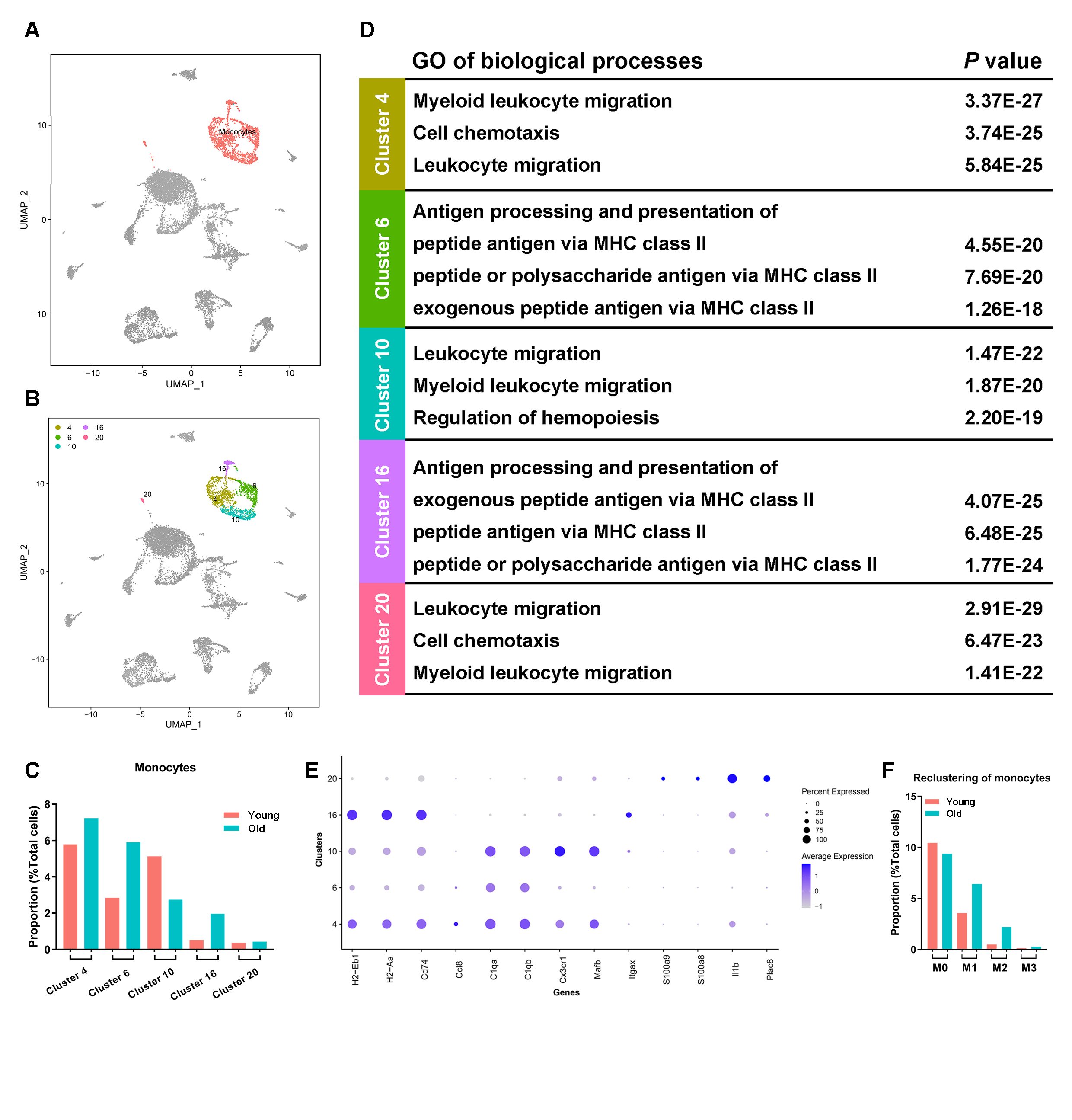

Supplement: Supplementary file 6 — Additional file 6: Supplementary Fig. 6 Single-cell features of monocytes. A. Uniform Manifold Approximation and Projection for Dimension Reduction (UMAP) plot of monocyte populations and their distribution in young and old samples (B). C. Cell proportions of monocyte clusters in young and old samples. D. The Gene Ontology (GO) terms of differentially expressed genes clusters with a fold change of log2 transformed unique molecular identifier > 1. E. Expression patterns of monocyte marker genes. The fraction of cells that expressed the marker genes is indicated by the size of the circle, and the means of the expression of marker genes are indicated by the color. F. Cell proportions of monocyte subclusters in young and old samples [file 12979_2023_345_MOESM6_ESM.jpg]
